# New Appliances and Things Medical

**Published:** 1909-05-29

**Authors:** 


### NEW APPLIANCES AND THINGS MEDICAL.

[We shall be glad to receive at our Office, 28 & 29 Southampton Street, Strand, London, W.C., from the manufacturers, specimens of all new preparations and appliances.]

### SCHERING'S MEDINAL.

(London Agent: A. and M. Zimmermann, 3 Lloyd's Avenue, E.C.)

The extensive use of veronal or diethylbarbituric acid has naturally led the manufacturers of synthetic remedies to try to improve upon this most useful hypnotic. Veronal, as all know who have used it, is insoluble. This means, pharmacologically, that both its absorption and elimination will probably be slow.

Clinical experience has shown, not so much that the onset of sleep is slow, as that the complete effects of veronal require some time to disappear: in other words, that the patient does not always pass rapidly from a restful sleep to a refreshed wakefulness, but that a period, more or less long, of drowsiness is apt to intervene between these two states.

The drug before us is the mono-sodium salt of diethylbarbituric acid, and it is soluble in water to the extent of 20 per cent. in the cold. Hence it is rapidly absorbed and rapidly eliminated, and is as suitable for administration by the rectum as by the mouth. It should be given by the stomach preferably three hours after the last meal, in doses of from 5 to 15 grains, previously dissolved in a wine-glassful of water. For rectal use 7½ grains should be given dissolved in about 1½ drachms of water. It may also be given hypodermically, in doses of from 7½ grains, dissolved in 1 drachm of water. Clinical experience has shown the efficacy of this drug, which may strengthen the hand of the physician in dealing with certain cases of insomnia.

